# On the Hygienè of Costume

**Published:** 1858-12

**Authors:** Amory Coffin

**Affiliations:** Aiken, South Carolina


### SELECTIONS.

*On the Hygienè of Costume.* BY AMORY COFFIN, M. D., of Aiken, South Carolina.

Dress, in its hygienic relations, certainly comes under the censorship of the physician, the natural guardian of the health of nations. In its fashions, its artistic effects, its outward appearance, it may be considered beneath our dignified notice: these aspect of dress may be safely left to Frank Leslie, Godey and other *dii minores*. To us, however, it belongs to consider *the effect of style of dress on health*; and when we find that a large and important class is subjected to a costume calculated to produce injurious results, it becomes our province, nay, our duty to raise our voices in solemn protest against it. Do not be alarmed, Mr. Editor, I am not going to open up again the oft argued question of corsets or hoops. Much has been said both for and against these articles of female dress, and they maintain their ground, the latter often doing more than this. I do not wish to attack a single article of dress, but a whole costume; and I verily believe that if some enemy of the human race had, with malice prepense, set himself to work to excogitate a dress which should surely and slowly produce an injurious effect on the growing organs of the rising generation, he could not, notwithstanding the traditional cunning of his class, have contrived one better adapted to his purpose than the full dress uniform of the various military schools now in existence in our State. Varying as they do in color, their construction, their build is the same. Charleston, if I remember, right, copied its dress from that of the old State soldiers, the predecessors of the cadets; Columbia follows Charleston, and Yorkville, and other military schools for boys, pursue the beaten track with admirably irrationality.

Let us take now the several components of this dress, *seriatim*, from top to toe, bearing in mind always as we go along, our latitude, that of Algeria, our summer temperature, the thermometer often rising above 90° Fahrenheit in the shade, and, what I will refer to hereafter, the age of the subjects of these injurious influences. First in order of these abominations comes the black leather shako, weighing, I don't know how many pounds, pressing with its hard, unyielding rim on the growing brain-case, interrupting the cranial, and indirectly the cerebral circulation, detaining black blood where it is not wanted, and where it is most injurious, and palpably exhibiting its effects in the fainting boys who are so often carried off from parade of a hot summer's day. To help in producing this effect, we have a perfectly impervious material, absolutely preventing any renovation of the heated, or, rather, *hot* air confined in the upper part. "To keep the head cool and the feet warm," is an old, but valuable hygienic piece of advice, perfectly disregarded, as far as the first part of it is concerned, in the adoption of this unsightly headgear. The only argument that can be adduced in its favor, is drawn from the protection it may afford the soldier, while on active duty, against sabre cuts directed at the head; but a similar reason might more plausibly be given for casing the arms in thick, heavy leather, as these limbs are more frequently wounded, in modern warfare, than the head. In fact, the whole accoutrement of the soldier, in modern times, is calculated more for aggression than defence, and the Minie cone would have very little more respect for the thick shako, than for the cloth cap or the sensible felt hat, which has recently been adopted in the United States Army. Nor is the prospect of this merely possible contingency a sufficient excuse for subjecting our youth to the probability of a life of headache or other cerebral disturbances. The incomplete protection to the eyes against the glare of the sun, or the irritation of cold winds, afforded by the small front-piece, is another objection to this senseless article, which has neither beauty, comfort nor utility to recommend it.

To assist in the injurious effects of the shako, we find the circulation of the carotids and jugulars impeded by the stock and high coat-color. Dr. Marshall has sufficiently shown the evil effects to be apprehended from any article of apparel that constringes the neck. I need not, therefore, dwell on it, but will refer your readers to his notes on the subject. I will not be croaker enough to talk about "hidden seizures, epileptic and apoplectic attacks;" but I must say, we could hit on no better plan to lay the foundation of, and predispose to them, than by this confinement of the throat.

The next nuisance that we encounter, proceeding downwards, is the padding of the breast of the coat. Setting aside the question whether it is not our duty on every occasion to teach youth to walk uprightly, and abhor and eschew *sham*, as belonging to the moral aspect of the case, I will confine myself to the hygienic point of view. Tubercles, we know, have their favorit seat at the summit of the lungs. This is, with much show of reasen, attributed to the less amount of expansion which this part of the cone undergoes during the physolo-gical act of respiration.

Indeed, some systems of treatment of consumption are based on this fact, and Dr. Muhry, in your March number, advises forced inspirations and expansion of the apex, as a prophylactic in tubercular predisposition.

What, then, shall we think of a style of dress which, at a period of life when this predisposition is so strongly developed, and a mere trifle may determine the question of robust health and enjoyment of life, or prolonged suffering and premature death, actually unnecesarily assists in promoting the production of disease, by the obstruction of this expansion. We cannot call it suicidal, because it is not voluntarily assumed. It might rather be classed with the Chinese method of artificially distorting young limbs, (of trees and children,) except that the organs practiced on are of vital importance, and the question becomes one of life or death, not of more or less beauty of deformity.

Two important circumstances we must bear in mind: the first is, that these deleterious influences are brought to bear upon their victims at a critical time of life. At this age the constitution is, as it were, beginning to crystalize. Earlier in life, time would still be left to recover from them. Later, after crystalization is completed, their influence would be much less. It is just at this period of adolescence that the seeds of much subsequent disease and suffering are shown.

The second is, that this is not the occasional holiday dress of the young clerk or mechanic. With them, the system has ample time to recruit. It is, in the case of the shako, the frequent, in the case of the other two, the every day attire of the cadet. Their influence is not intermittent, but continued.

Having thus pointed out the defects and evil influences of this costume, I may be asked what I would substitute for it. Here, as in other cases, I must confess, criticism is easy; construction is difficult.

But yet, I thing, having pointed out the dangers to be avoided, a dress might be constructed which, without being too fantastic,—not more so, certainly, than the present unpicturesque uniform, which gives them the air of playing at soldiers,—would not press heavily on the head, obstruct the circulation of the neck, nor impede the respiration at the very part where it is most important it should be fullest and most expanded.

The felt hat of the United States Army, light and serviceable, protecting both head and eyes; the neck free from stock and stiff coat-collar, but showing the commencement of the shirt; a long, loose jacket, somewhat like that worn by the Zouaves, extending below the hips, but allowing full play to the muscles of the femur, with rather short, loose, sleeves, to give the arms room for action and development; a broad belt around the waist, to support the diaphragm and throw the burthen of respiration on the upper part of the lungs, thereby causing expansion of the thorax; rather loose trowsers, extending not much below the ankle, so as not to interfere with the play of that articulation; with, if you choose, shoes and gaiters. These ingredients might, with more or less ornament, according to taste, be concocted by a skillfull artist into a healthful and becoming dress for the cadets of our military schools.

These institutions, from their admirable discipline, perhaps also as foreshadowing coming events, are becoming very popular, and it is important that anything which would detract from their utility should be corrected or avoided; and where it is a question of health or hygienè the persons to point out these dangers are the physicians of the State.—*Charleston Medical Journal.*

